# Stable Signal Peptides and the Response to Secretion Stress in *Staphylococcus aureus*

**DOI:** 10.1128/mBio.01507-17

**Published:** 2017-12-12

**Authors:** Arryn Craney, Floyd E. Romesberg

**Affiliations:** Department of Chemistry, The Scripps Research Institute, La Jolla, California, USA; McMaster University

**Keywords:** *Staphylococcus aureus*, secretion systems, signal peptidase, stress response, transcriptional regulation

## Abstract

Protein secretion is essential, but how it is managed is poorly understood. In bacteria, most secreted proteins require release from the outer surface of the cytoplasmic membrane by type I signal peptidase (SPase), which cleaves the mature protein from its membrane-bound N-terminal signal peptide. As the first step that occurs outside the protected cytoplasmic environment and because insufficient activity can rapidly result in the toxic accumulation of preproteins, the activity of SPase is expected to be closely monitored and perhaps supplemented when insufficient. Indeed, we previously demonstrated that inhibition of SPase in *Staphylococcus aureus* results in derepression of the *ayrRABC* operon, which encodes an alternate mechanism to release proteins. However, in this case, the proteins are released with partially intact signal peptides, with the exception of IsaA, which is released with a virtually intact signal peptide. Here we show that mutation of AyrA [*ayrA*(R233K)] results in constitutive derepression of *ayrRABC* and that mutation of IsaA’s signal peptide [*isaA*(K2Q)] results in hyperderepression upon SPase inhibition, which also requires AyrA. Further studies demonstrate that the inducing signal for *ayrRABC* derepression is accumulation of a subset of preproteins with signal peptides that are stable toward further processing and that the signal is critically amplified by the K2Q mutation and relayed to AyrR by AyrA. These results elucidate the mechanism by which *S. aureus* monitors and responds to secretion stress. The presence of *ayrRA* in other bacteria suggests that it may represent a general strategy linking membrane stress to appropriate transcriptional responses.

## IMPORTANCE

Bacteria interact with their environment by secreting proteins that perform a myriad of functions, and the final step is the release of the mature protein from the cell surface via the activity of type I signal peptidase (SPase). While the bacterial response to many stresses is understood in some detail, almost nothing is known about how cells respond to secretion stress, such as insufficient SPase activity, which would eventually result in cell death. We previously demonstrated that the inhibition of SPase in *Staphylococcus aureus* results in the derepression of the *ayrRABC* operon, which can functionally replace SPase, but which is normally repressed by AyrR. We now demonstrate that the inducing signal for derepression is accumulation of a subset of preproteins with signal peptides that are stable to further processing and that the signal is relayed to AyrR via AyrA.

## OBSERVATION

Protein secretion is an essential part of the physiology and virulence of all bacteria, and while inefficient secretion is thought to represent a significant stress, the inducing signal(s) and mechanism(s) by which the cell responds to tolerate or eliminate the stress are largely unknown. Under normal conditions, a critical step in the secretion of most proteins is their release from the extracellular face of the cytoplasmic membrane by type I signal peptidase (SPase) (1). SPase catalyzes the proteolytic release of the mature protein from the N-terminal signal (or leader) peptide that directed it to the general secretory (Sec) or twin arginine translocation (TAT) pathway for translocation across the cytoplasmic membrane (2). Once the mature protein is released, the signal peptide is thought to be proteolyzed by a site 2 protease (3), which leads to its removal from the membrane.

**FIG 1:**
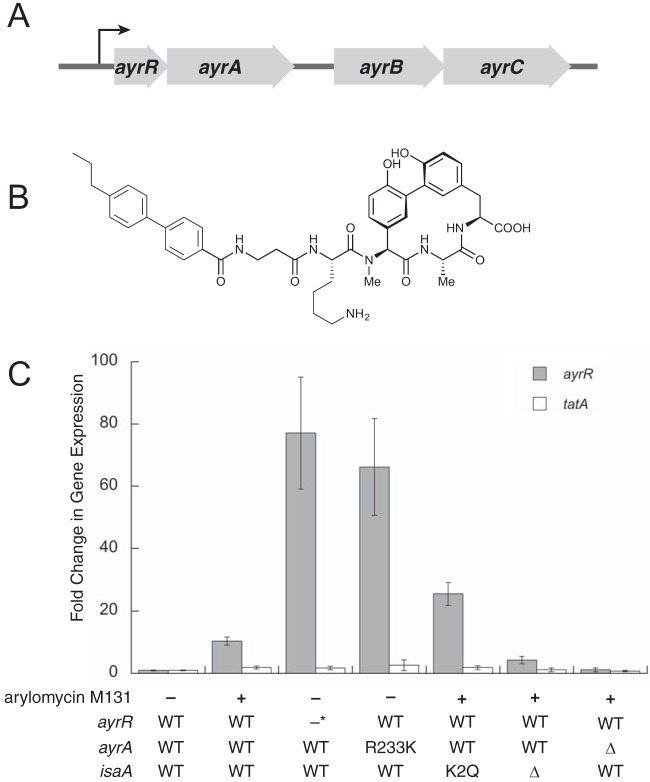
Induction of the *ayrRABC* operon. (A) Illustration of the *ayrRABC* operon (B) Chemical structure of arylomycin M131. (C) Induction of *ayrRABC* followed by RT-PCR with or without arylomycin M131 treatment in strains harboring wild-type (WT), deletion (Δ), or mutant forms of *ayrR*, *ayrA*, or *isaA* as indicated (with an asterisk denoting a loss-of-function mutant [5]). Strains were grown in tryptic soy broth (TSB) to an OD_600_ of 1.0 followed by RNA extraction and cDNA synthesis, except for strains requiring arylomycin M131 treatment for induction of *ayrRABC*, which instead were grown to an OD_600_ of 0.6, treated with 4 µg/ml arylomycin M131, and then grown to a final OD_600_ of 1.0 (total treatment time of 20 min) before continuing with RNA extraction and cDNA synthesis. cDNA was subjected to RT-PCR using primers specific for *ayrR* to measure expression of *ayrRABC* and for *tatA* as an external control. Gene expression was normalized using *gmk* and compared to that in the untreated parental strain, *S. aureus* N315. Fold changes in gene expression were calculated using the threshold cycle (ΔΔ*C*_*T*_) method; the data shown are the average and standard error of the mean (SEM) from three independent samples. The *P* values for *ayrR* expression in the mutant strains relative to the wild-type uninduced sample were as follows: *P* < 0.005 for N315 and the *isaA*(K2Q) mutant with arylomycin addition, *P* < 0.005 for the *ayrR* loss-of-function mutant and *ayrA*(R233K) mutants, *P* < 0.01 for the Δ*isaA* mutant with arylomycin addition, and *P* > 0.05 for the Δ*ayrA* mutant with arylomycin addition. The *P* values for *tatA* expression in mutant strains relative to the wild-type uninduced sample were as follows: *P* > 0.025 for N315 and *P* > 0.05 for the *isaA*(K2Q) Δ*isaA* and Δ*ayrA* mutants, all with arylomycin addition, and *P* > 0.05 for the *ayrR* loss-of-function mutant and *ayrA*(R233K) mutants.

Previously in *Staphylococcus aureus*, we showed that the inhibition of SPase by arylomycin M131, a semisynthetic member of the arylomycin class of natural products (4), results in derepression of the *ayrRABC* operon (Fig. 1A), whereas under normal conditions constitutively expressed AyrR binds upstream of its own gene and represses expression of the operon (5, 6). Moreover, we showed that derepression of *ayrRABC* bypasses the need for SPase, because the operon encodes proteins that constitute an alternate mechanism to release proteins from the cytoplasmic membrane, results later confirmed by Morisaki et al. (7). We speculated that AyrRABC acts as a backup system to mediate the release of proteins when the activity of SPase is inhibited or simply insufficient. While AyrRABC is able to compensate for the loss of SPase, its activity results in the release of proteins with partially intact signal peptides (6, 7), cleaved not at the SPase cleavage site, but rather within the signal peptide itself (7). A single exception observed was IsaA, which was detected with an intact or at least nearly intact signal peptide (6). While it is unclear if the intact or partially intact signal peptides affect the function of the secreted proteins, the induction of the operon clearly mediates resistance to SPase inhibition by preventing the toxic accumulation of the preproteins in the membrane.

AyrB and AyrC appear to constitute the ATP-binding cassette and transporter domains, respectively, of a type 2 family ABC transporter (6). Interestingly, another bacterial ABC transporter, LolCDE, has been shown to extract lipid-anchored proteins from the cytoplasmic membrane (8), and we speculate that AyrBC serves a similar function with proteins anchored by intact or partially intact signal peptides. Indeed, mutations that have been shown to prevent ATP binding in other ABC transporters abolish resistance to SPase inhibition when introduced into AyrB (7). The possible function of AyrA, a predicted polytopic membrane protein with a domain of unknown function (DUF3169/PF11368), is less clear. AyrA is not required for resistance to SPase inhibition if the operon is derepressed (7), suggesting that it might be involved in derepression. Homologous proteins of the DUF3169 family are found in both staphylococcal and streptococcal species, and while their sequence identity is only ~30%, they all contain six putative transmembrane domains and a conserved D-E(a/g)E sequence located in the loop connecting the fourth and fifth predicted transmembrane segments.

As part of an effort to understand the response to secretion stress in bacteria and the role of the AyrRABC system in *S. aureus* in particular, we sought to identify mutations that confer resistance to arylomycin M131, but which are not located in the repressor AyrR, where loss-of-function mutations provide an obvious and already characterized route to *ayrRABC* induction (5–7). Two classes of mutations were identified: one in AyrA and one in the signal peptide of IsaA. Characterization of the mutations suggests that both mechanisms of resistance ultimately function via *ayrRABC* derepression, that the inducing signal is the accumulation of a subset of intact preproteins with signal peptides that are atypically resistant to processing by site 2 proteolysis, and that the signal is likely relayed to AyrR via AyrA. These results elucidate a novel mechanism to detect and manage secretion stress in *S. aureus*.

### Mutations in *ayrA* or the signal peptide of IsaA confer resistance to SPase inhibition via a mechanism that requires *ayrRABC*.

The *ayrRABC* operon was identified based on its induction by arylomycin M131 (Fig. 1B) (5, 6), and by the identification of *ayrR* loss-of-function mutations that confer high-level resistance to arylomycin M131 (MIC increase from 1 μg/ml to >128 μg/ml) through constitutive derepression of the operon (6). However, as originally noted (5), only ~75% of the resistance-conferring mutations identified occur within *ayrR*. Thus, to further elucidate how the *ayrRABC* operon is regulated, we evolved additional mutants of *S. aureus* N315 that are resistant to SPase inhibition (16 μg/ml arylomycin M131), but which retain a wild-type *ayrR* gene. Sequencing the entire *ayrRABC* operon of resistant isolates identified two classes of mutations, each conferring high-level resistance (MIC of >128 μg/ml [see Table S1 in the supplemental material]). The first class encoded an Arg-to-Lys mutation at position 233 of AyrA. A full restoration of arylomycin sensitivity was observed with this class of mutant upon deletion of *ayrBC* (MIC of 1 μg/ml [Table S1]), confirming that its mechanism of resistance is fully dependent on AyrBC. Furthermore, *ayrA*(R233K) was found to confer constitutive expression of *ayrRABC* at levels similar to those observed in the *ayrR* loss-of-function mutant, and deletion of *ayrA* results in the inability to induce the *ayrRABC* operon (Fig. 1C). This, along with its membrane location, suggests that AyrA may be involved in converting the signal for secretion stress into derepression of *ayrRABC*, possibly through a direct interaction with AyrR. The location of the R233K mutation, predicted to be at the interface of the membrane and the cytoplasm (see Fig. S1 in the supplemental material), may facilitate this function. A functional interaction between AyrA and AyrR is also supported by a survey of staphylococcal and streptococcal species, which reveals that *ayrA* homologues are virtually always present immediately downstream of *ayrR* homologues, although *ayrBC* homologues are often not present (see Fig. S2 in the supplemental material).

10.1128/mBio.01507-17.2 FIG S1  Dot diagram of AyrA illustrating the predicted location of the R233K mutation and IsaA illustrating the location of the K2Q mutation (both mutations shown in red). The membrane topologies shown are based on the TMHMM server v.2.0 (http://www.cbs.dtu.dk/services/TMHMM/). For simplicity, only the signal peptide portion of IsaA is depicted. Download FIG S1, PDF file, 0.4 MB. Copyright © 2017 Craney and Romesberg. 2017 Craney and Romesberg This content is distributed under the terms of the Creative Commons Attribution 4.0 International license.

10.1128/mBio.01507-17.3 FIG S2  Organization of *ayrRA* and downstream genes in various staphylococcal and streptococcal strains. Download FIG S2, PDF file, 0.3 MB. Copyright © 2017 Craney and Romesberg. 2017 Craney and Romesberg This content is distributed under the terms of the Creative Commons Attribution 4.0 International license.

10.1128/mBio.01507-17.4 TABLE S1  Arylomycin M131 MIC values. Download TABLE S1, PDF file, 0.1 MB. Copyright © 2017 Craney and Romesberg. 2017 Craney and Romesberg This content is distributed under the terms of the Creative Commons Attribution 4.0 International license.

The second class of resistant mutants isolated retained an entirely wild-type *ayrRABC* operon, and full genome sequencing of one mutant revealed a single mutation in IsaA, K2Q, which is located in the protein’s signal peptide (Fig. S1). The resistance to SPase inhibition conferred by *isaA*(K2Q) was fully ablated by deletion of *ayrRABC* (arylomycin M131 MIC of 1 μg/ml [Table S1]), demonstrating that its mechanism of resistance is fully reliant upon AyrRABC.

### Neither altered production nor altered SPase recognition is responsible for resistance mediated by IsaA(K2Q).

Based on its presence in a SPase substrate, we speculated that the K2Q mutation of IsaA may be related to the inducing signal for the secretion stress response. For example, if the processing of IsaA represents a particular burden on SPase, or if once translocated the activity of IsaA itself is deleterious if not processed by SPase, reduced translocation of IsaA(K2Q) via Sec could contribute to the observed resistance. To examine this possibility, we first constructed an N315 Δ*isaA* strain, which we found has only a modest level of resistance to SPase inhibition (arylomycin M131 MIC of 8 μg/ml [Table S1]).

To determine whether the modest 8-fold decrease in sensitivity conferred by the deletion of *isaA* results simply from a reduction in the amount of preprotein that requires SPase for processing or from something more specific to the loss of IsaA, we examined the effects of ectopic expression of BlaZ from the *isaA* promoter (P*_isaA_-blaZ*). As a control, we first examined ectopic expression of *isaA* from its native promoter (P_*isaA*_-*isaA*) in N315 Δ*isaA* and found that it restored full sensitivity (MIC of 1 μg/ml [Table S1]). Expression of the P*_isaA_-blaZ* construct also resensitized Δ*isaA* cells to arylomycin M131 (MIC of 2 μg/ml). Thus, ectopic expression of either IsaA or BlaZ from the *isaA* promoter in the Δ*isaA* strain results in similar, wild-type-like sensitivity to SPase inhibition, suggesting that the 8-fold-increased resistance of the Δ*isaA* strain relative to its parental wild-type strain results from a general reduction of the burden on SPase. As expected, ectopic expression of P_*isaA*_-*isaA*(K2Q) resulted in high-level resistance to SPase inhibition (arylomycin M131 MIC of >128 μg/ml [Table S1]). Thus, while native expression of IsaA introduces a modest burden on SPase, the elimination of IsaA does not account for the high-level resistance mediated by the IsaA(K2Q) mutation.

**FIG 2:**
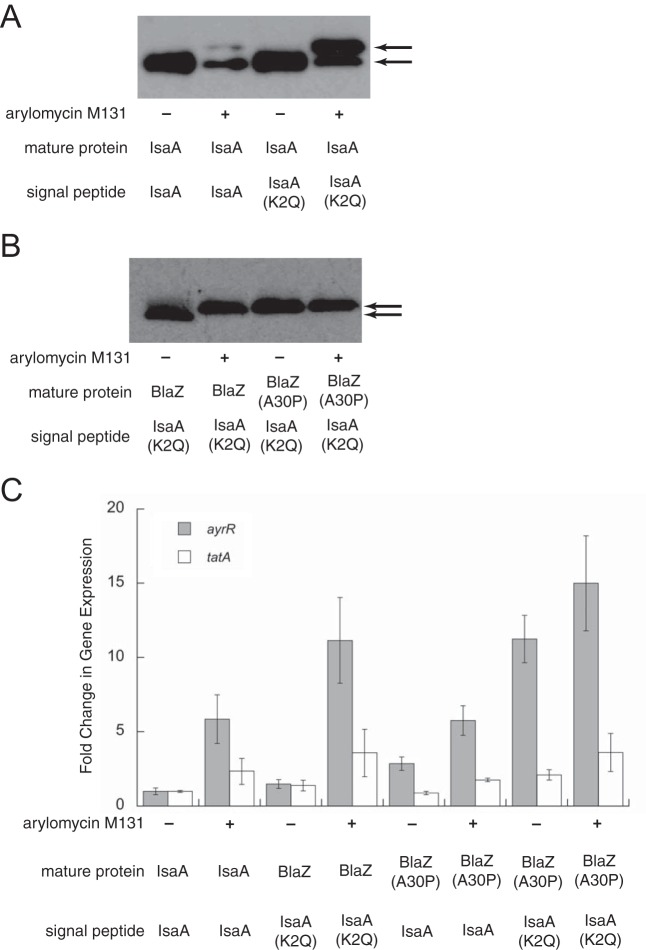
Effects of the K2Q mutation and the stability of the signal peptide. (A) Western blot comparing secretion of IsaA with or without the K2Q mutation. Arrows indicate positions of the two differently migrating bands (see text for details). (B) Western blot comparing secretion of hybrid constructs consisting of the IsaA(K2Q) signal peptide appended to the mature protein domain of BlaZ with or without the A30P mutation. Arrows indicate positions of the two differently migrating bands (see text for details). For panels A and B, strains were grown in TSB with chloramphenicol and collected at an OD_600_ of 0.6, and the resulting cell pellet was washed twice in sterile saline to remove previously secreted proteins. Cell pellets were resuspended to an OD_600_ of 0.6 and split, with one culture receiving dimethyl sulfoxide (DMSO) and one receiving 4 µg/ml arylomycin M131. Cultures were grown for 30 min and centrifuged, and the supernatant corresponding to the secreted fraction was subject to trichloroacetic acid (TCA) precipitation and then analyzed by Western blotting using a horseradish peroxidase (HRP)-conjugated primary antibody directed against the 6×His tag. (C) Induction of the *ayrRABC* operon. Briefly, cultures were grown in TSB plus chloramphenicol to an OD_600_ of 0.6 and then split, with one receiving DMSO (indicated by +) and the other receiving 4 µg/ml arylomycin M131 (indicated by −). Cultures were grown for an additional 20 min followed by RNA extraction and cDNA synthesis. cDNA was subject to RT-PCR using primers to amplify *ayrR* or *tatA* as a control. Gene expression was normalized using *gmk* and compared to the plasmid-borne *isaA* construct P_*isaA*_-*isaA*. Fold changes in gene expression were calculated using the ΔΔ*C*_*T*_ method; the data shown are the average and standard error of the mean (SEM) from three independent samples. The *P* values for *ayrR* expression in strains relative to the plasmid-borne *isaA* construct P_*isaA*_-*isaA* uninduced control were as follows: *P* < 0.01 for all strains, with the exception of uninduced P_*isaA*_-*isaA*(K2Q)_SP_*blaZ*_EC_, which was *P* > 0.05. The *P* values for *tatA* expression in strains relative to the P*_isaA_-isaA* uninduced control were as follows: *P* > 0.025 for all except P_*isaA*_-*isaA*_SP_*blaZ*_EC_ with arylomycin and *isaA*_SP_*blaZ*(A30P)_EC_ without arylomycin (*P* < 0.01). In the construct designations, subscript SP represents the predicted signal peptide and subscript EC represents the predicted mature protein.

The IsaA(K2Q) mutation may mediate resistance by altering the recognition of the IsaA preprotein by SPase. To directly assess the effect of the K2Q mutation on SPase processing, we compared the secretion of ectopically expressed and C-terminally His-tagged IsaA and IsaA(K2Q) in N315 Δ*isaA*. Cells were grown to on optical density at 600 nm (OD_600_) of 0.6, washed, and then incubated with or without 4 μg/ml arylomycin M131 for 30 min, and the presence of IsaA or IsaA(K2Q) in the medium was analyzed by Western blotting with an anti-His antibody (Fig. 2A). No differences in secretion were observed between IsaA and IsaA(K2Q) in the absence of arylomycin. However, in the presence of the arylomycin, we observed greater secretion of IsaA(K2Q) than IsaA, and in both cases, two protein bands were observed—one corresponding to the normally secreted IsaA protein and one migrating more slowly that was dramatically more abundant with IsaA(K2Q). The faster-migrating band likely originates from residual SPase activity. The slower-migrating band is identical to that observed previously with the wild-type IsaA protein with SPase inhibition and which was shown to result from AyrRABC-mediated secretion of IsaA with an N-terminal signal peptide that was intact or at least nearly intact (6). Thus, while the K2Q mutation allows for continued secretion of IsaA, albeit with an intact signal peptide, it does not alter the protein’s production or its recognition by SPase when SPase is not inhibited.

### The IsaA(K2Q) mutation induces derepression of *ayrRABC*.

The increased secretion of IsaA(K2Q) relative to IsaA when SPase is inhibited, as well as its secretion with an at least mostly intact signal peptide, suggests that the K2Q mutation may mediate resistance by inducing increased *ayrRABC* derepression. Indeed, transcriptional analysis following treatment with 4 μg/ml arylomycin M131 revealed a significant increase in expression of *ayrRABC* in the IsaA(K2Q) strain relative to the wild type, whereas complete deletion of *isaA* did not have a significant effect (Fig. 1C). Unlike AyrA(R233K), the increased derepression of *ayrRABC* with IsaA(K2Q) was observed only with SPase inhibition. The data suggest that the accumulation of the intact IsaA(K2Q) preprotein acts to signal the derepression of *ayrRABC*.

### IsaA(K2Q)-mediated resistance to SPase inhibition requires a stable signal peptide or preprotein that is not processed by SPase.

Ravipaty and Reilly reported an analysis of free signal peptides found in the medium fraction of an actively growing culture of *S. aureus* COL (9). As expected, the majority of the recovered signal peptides were found as fragments, presumably resulting from cleavage within the signal peptide by a site 2 protease (possibly RseP/SA1105 [3]), after liberation from the mature protein by SPase. However, five signal peptides, including that of IsaA, were recovered intact (i.e., without site 2 cleavage; here we refer to these as stable signal peptides). Thus, we next explored the relationship between the K2Q mutation and the stability of the signal peptide, assuming that the stability of the signal peptide was independent of its associated mature protein. We first constructed plasmids expressing the mature IsaA protein fused to the signal peptide from SceD (also found to be stable by Ravipaty and Reilly [9]), or the signal peptide from SA1574 (which does not appear to be stable [6, 7]). In both cases, the preproteins were ectopically expressed in N315 Δ*isaA*, with or without a K2Q mutation. (Both native signal peptides have a Lys at position 2.) We observed high-level arylomycin resistance with the K2Q mutant of the SceD (stable) signal peptide (MIC of >128 μg/ml [Table S1]), but not with K2Q mutant of the SA1574 (unstable) signal peptide (MIC of 1 μg/ml).

To explore this effect further, we compared the effects of ectopic expression of the mature BlaZ protein fused to its own signal peptide with the K2Q mutation or fused to the IsaA signal peptide with the K2Q mutation. The K2Q mutation again conferred high-level arylomycin resistance with the IsaA signal peptide (MIC of >128 μg/ml [Table S1]), but not with the BlaZ signal peptide (MIC of 1 μg/ml). As a final control, we constructed a plasmid expressing a variant of the N-terminal transmembrane domain of AtpF with an N-terminal MQK tripeptide extension fused to the IsaA extracellular domain. AtpF lacks a signal peptide, but the MQK-AtpF construct should localize to the membrane with Gln2 at the cytoplasm-membrane interface, similar to the position of Gln2 in IsaA(K2Q). No resistance was observed with expression of this construct (arylomycin M131 MIC of 1 μg/ml), consistent with a mechanism of resistance associated with the Gln2 residue that requires the context of a (stable) signal peptide.

To unambiguously determine whether the K2Q mutation requires a stable signal peptide, or if inhibition of SPase processing is sufficient, we transformed the Δ*isaA* strain with a plasmid expressing a hybrid IsaA-BlaZ construct composed of the IsaA signal peptide with or without the K2Q mutation fused to the extracellular domain of BlaZ with a C-terminal His tag. The construct also has the amino acid immediately C terminal to the SPase cleavage site mutated to Pro (A30P) to prevent SPase cleavage, as has been shown for an analogous mutation in other signal peptides (10–12), and which was confirmed in this case via Western blotting (Fig. 2B). Cells expressing the various IsaA-BlaZ constructs were then grown under identical conditions, with or without added arylomycin M131 (4 μg/ml), and transcription of the *ayrRABC* operon was characterized by reverse transcription (RT)-PCR (Fig. 2C). Control strains with either the wild-type IsaA or the IsaA(K2Q) mutant signal peptide fused to the BlaZ extracellular domain only induced *ayrRABC* derepression upon arylomycin addition. With the native Lys2 IsaA-BlaZ(A30P) construct, a low level of induction was observed in the absence of the arylomycin, and derepression was increased somewhat with its addition, likely due to the accumulation of other preproteins. However, with the IsaA(K2Q)-BlaZ(A30P) construct, the operon was already much more strongly induced in the absence of arylomycin treatment and increased only slightly with arylomycin treatment, again likely due to the accumulation of other preproteins. Thus, the high-level operon derepression mediated by the K2Q mutation requires the context of a stable signal peptide, even if processing of the preprotein by SPase is inhibited.

### Conclusions and a model for the *S. aureus* response to secretion stress.

Under normal growth conditions, SPase mediates the proteolytic release of mature proteins, and the remaining signal peptide is then thought to be internally proteolyzed, which then facilitates its elimination from the membrane. This internal cleavage is thought to be mediated by a so-called “site 2 protease,” possibly RseP/SA1105 in *S. aureus* (3), and this activity is thought to be generally less efficient in the absence of so-called “site 1 cleavage” by SPase (3, 13, 14). We speculate that with most signal peptides under normal conditions, site 2 cleavage remains efficient enough to proceed at a sufficient rate, even in the absence of SPase cleavage (for a review of emerging evidence that site 2 cleavage can occur in the absence of site 1 cleavage, see reference 15). However, it has been known for years that some signal peptides are more resistant to cleavage (16–18), and in *S. aureus*, this includes IsaA and SceD (9). When the efficiency of their site 2 cleavage is reduced even further through inhibition of the SPase-mediated site 1 cleavage step, and when expressed at sufficient concentrations, these persistent preproteins act as the signal of secretion stress. The accumulation of even one of these preproteins acts as a potent signal for *ayrRABC* derepression. We further speculate that during normal growth, the capacity of SPase may occasionally be saturated, and this may result in the accumulation of sufficient levels of intact preproteins to induce *ayrRABC* derepression. Thus, the secretion stress response is not strongly induced by the accumulation of preproteins in general, but rather by the accumulation of a small subset of preproteins that possess a stable signal peptide. Within the complete secretome of *S. aureus*, there appear to be five such preproteins (9); however, IsaA might be a particularly potent signal when SPase is inhibited because it is one of the most highly expressed proteins in *S. aureus* (19–21), likely explaining why IsaA mutants were isolated in our screen for clones resistant to SPase inhibition.

The basis for our model is the operon derepression caused by the K2Q mutation and its dependence on the stability of the signal peptide and the processing of the associated preprotein by SPase. We suggest that the K2Q mutation may amplify the signal induced by the preprotein, perhaps by facilitating interactions with a downstream protein or simply by further increasing stability toward site 2 cleavage. Consistent with the latter possibility, the most stable signal peptide identified by Ravipaty and Reilly was that of SACOL0507 (which was detected in its intact state only) (9), and the second residue of this signal peptide is naturally a Gln. However, if this is true, SACOL0507 must not be expressed at sufficient levels to induce *ayrRABC* derepression (as otherwise the wild-type strain would be resistant to SPase inhibition). Consistent with its insufficient expression, we did not detect the protein in the medium fraction of an *S. aureus* culture (6).

A likely candidate for a downstream protein that conveys the signal arising from the accumulation of preproteins to *ayrRABC* derepression is AyrA, as its presence is required for the IsaA(K2Q) mutation to exert its effect. Perhaps when activated by the accumulation of intact preproteins, AyrA may be converted to a form that cleaves, sequesters, or otherwise inhibits the ability of AyrR to repress *ayrRABC*, and this activated state may also be induced or mimicked by the R233K mutation, which we found results in the constitutive derepression of the operon. Future efforts will focus on further understanding the function of AyrA and testing this model. Finally, the presence of *ayrRA* homologues in other species, but without *ayrBC* homologues, suggests that AyrR and AyrA might represent a novel two-component signaling system that couples different membrane signals to other transcriptional responses.

10.1128/mBio.01507-17.1 TEXT S1  Supplemental methods. Download TEXT S1, PDF file, 0.1 MB. Copyright © 2017 Craney and Romesberg. 2017 Craney and Romesberg This content is distributed under the terms of the Creative Commons Attribution 4.0 International license.
