# Supplementary material for: Stable Signal Peptides and the Response to Secretion Stress in Staphylococcus aureus
Source: mBio. 2017 Dec 12;8(6):e01507-17. doi: 10.1128/mBio.01507-17 (PMC5727409; doi:10.1128/mBio.01507-17)
Supplement: FIG S1 [file mbo006173624sf1.pdf]

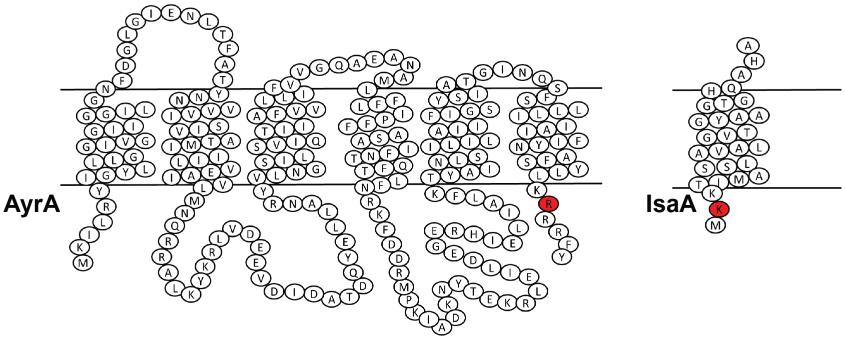

Figure S1. Dot diagram of AyrA illustrating the predicted location of the R233K mutation and IsaA illustrating the location of the K2Q mutation (both mutations shown in red). For simplicity only the signal peptide portion of IsaA is depicted.
