# Supplementary material for: Stable Signal Peptides and the Response to Secretion Stress in Staphylococcus aureus
Source: mBio. 2017 Dec 12;8(6):e01507-17. doi: 10.1128/mBio.01507-17 (PMC5727409; doi:10.1128/mBio.01507-17)
Supplement: FIG S2 [file mbo006173624sf2.pdf]

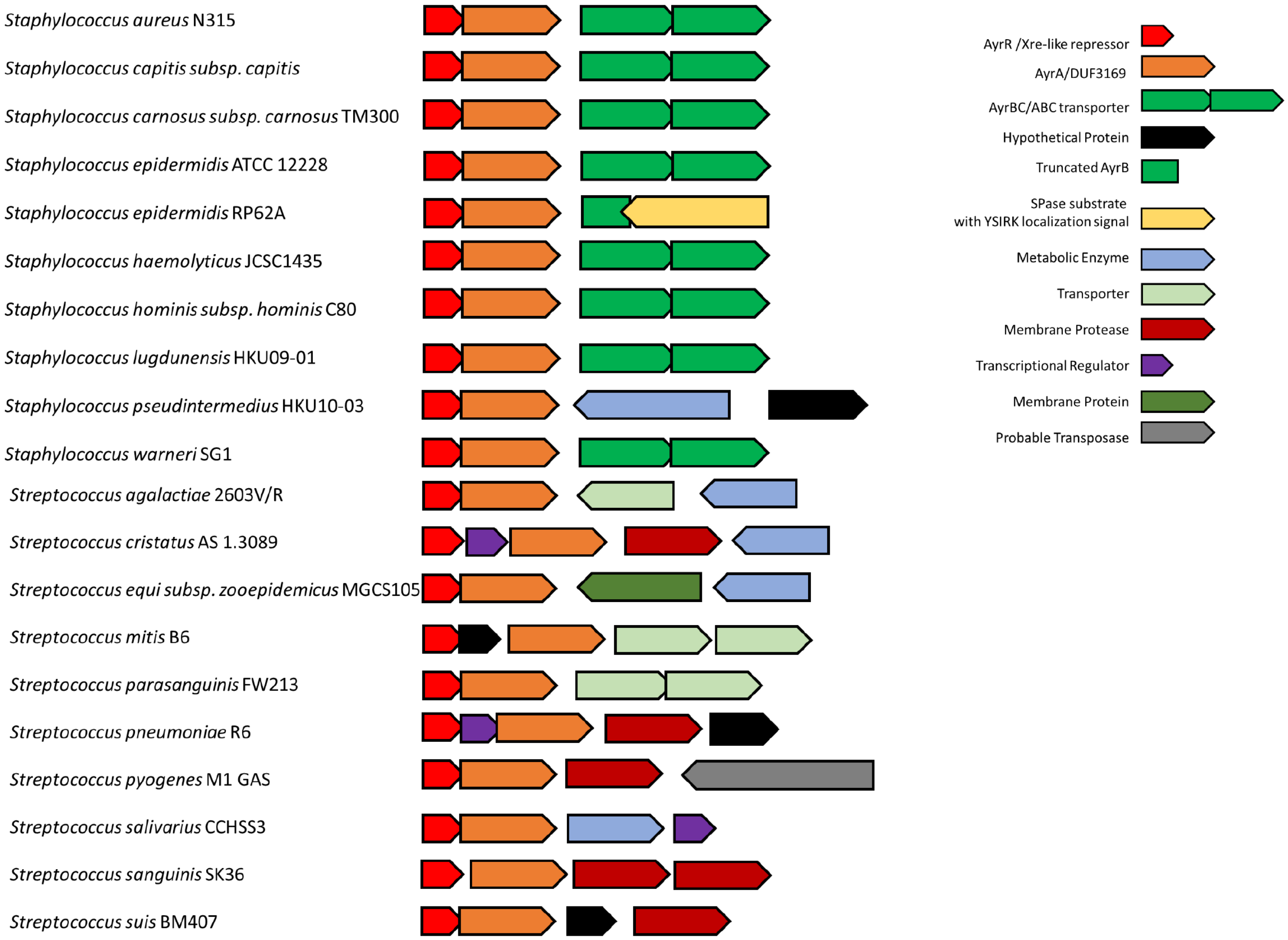

Figure S2. Organization of *ayrRA* and downstream genes in various Staphylococcal and Streptococcal strains.
