# Supplementary material for: Stable Signal Peptides and the Response to Secretion Stress in Staphylococcus aureus
Source: mBio. 2017 Dec 12;8(6):e01507-17. doi: 10.1128/mBio.01507-17 (PMC5727409; doi:10.1128/mBio.01507-17)
Supplement: TABLE S1 [file mbo006173624st1.pdf]

Table S1

Strain	Arylomycin M131 MIC (μg/ml)
N315	1
ayrR(L4X)	>128
ayrA(R233K)	>128
ayrA(R233K)Δ*ayrBC*	1
isaA(K2Q)	>128
isaA(K2Q)Δ*ayrRABC*	1
isaA(K2Q)Δ*isaA*	8
N315Δ*isaA*	8
N315Δ*isaA*Δ*ayrA*	8
N315Δ*isaA* + P_{isaA} - *isaA*	1
N315Δ*isaA* + P_{isaA} - *isaA*(K2Q)	>128
N315Δ*isaA* + P_{isaA} - *blaZ*	2
N315Δ*isaA* + P_{isaA} - *blaZ*(K2Q)	2
N315Δ*isaA* + P_{isaA} - *isaA*$(K2Q)_{SP}$*blaZ*$_{EC}$	>128
N315Δ*isaA* + P_{isaA} - *atpF*$(K2Q)_{SP}$*isaA*$_{EC}$	2
N315Δ*isaA* + P_{isaA} - $SA1754_{SP}$*isaA*$_{EC}$	1
N315Δ*isaA* + P_{isaA} - $SA1754(K2Q)_{SP}$*isaA*$_{EC}$	1
N315Δ*isaA* + P_{isaA} - *sceD*$_{SP}$*isaA*$_{EC}$	1
N315Δ*isaA* + P_{isaA} - *sceD*$(K2Q)_{SP}$*isaA*$_{EC}$	>128

SP refers to the predicted signal peptide, EC refers to the predicted extracellular region
