# Supplementary material for: Stable Signal Peptides and the Response to Secretion Stress in Staphylococcus aureus
Source: mBio. 2017 Dec 12;8(6):e01507-17. doi: 10.1128/mBio.01507-17 (PMC5727409; doi:10.1128/mBio.01507-17)
Supplement: TEXT S1 [file mbo006173624s1.pdf]

Material and Methods

Bacterial strains, growth conditions and antibiotic susceptibility testing. *S. aureus* N315 was cultured using tryptic soy broth (TSB) or tryptic soy agar (TSA) at 37 °C. Antibiotics ampicillin and chloramphenicol were used for plasmid selection at a concentration of 100 µg/ml and 20 µg/ml, respectively. Arylomycin M131 was synthesized as previously described (1, 2) and used at a concentration of 4 µg/ml corresponding to 4× the MIC of wild type *S. aureus* N315. Susceptibility to arylomycin M131 was performed as previously described (3). For strains containing plasmids, susceptibility testing was performed in the presence of chloramphenicol for plasmid selection. Evolved arylomycin-resistant isolates were obtained as previously described (3).

Whole genome sequencing of an evolved arylomycin M131 resistant isolate of *S. aureus* N315. Approximately 570 ng total RNA was fragmented using a Covaris S2 ultrasonicator (5% duty cycle, Intensity = 3, 200 cycles per burst, 40 s). Fragmented DNA was cleaned up using 0.5× Ampure beads and eluted in 0.1× TE. DNA was then prepared into a sequencing library using the New England Biolabs NEBNext® Ultra™ DNA Library Prep Kit for Illumina following the manufacturer's instructions with 6 cycles of PCR. The library was then sequenced on an Illumina MiSeq using 2 × 300 paired-end reads.

Strain construction. The *isaA* deletion strain was created as previously described (4). Briefly, a ~1 kb region upstream of the *isaA* gene was PCR amplified using primers IsaA_Up_For (5'-GGAGGTACCGCAGTATTGATAATTGGTACA) and IsaAUp_Rev (5'-ACATTACTTTTATTTATTATGAAGGAATTACATAGTAAAAAATCCTCCAGT). A ~1 kb region downstream of the *isaA* gene was PCR amplified using primers IsaA_DWN_For (5'-ACAATTATTACTGGAGGATTTTTTACTATGTAATTCCTTCATAATAAATAAAAG) and IsaA_DWN_Rev (5'- TCCGGAATTCGTAACAGAATCATTAAGATATGC). The upstream and downstream PCR products were amplified using IsaA_UP_For and IsaA_DWN_Rev to create a ~2 kb product using overlap extension. The 2 kb product was introduced into pIMAY by restriction digest (KpnI and EcoRI) and ligation. The resulting deletion plasmid was transformed into SA30B (Lucigen, Wisconsin USA). 4 µg of DNA isolated from SA30B was used to transform the deletion plasmid into *S. aureus* N315 by electroporation as previously described (3) and plated on TSB plus chloramphenicol at 30 °C. Single recombination of the deletion plasmid was achieved by subculturing 1:100 in TSB plus chloramphenicol at 43 °C for 24 h twice, followed by plating on prewarmed TSA plus chloramphenicol at 43 °C. For the second recombination of the plasmid, a single colony was inoculated in TSB without selection, grown for 24 h and subcultured 1:100 repeatedly until plasmid loss occurred. Plasmid loss was assessed by plating on TSA without selection and patching colonies on TSA with and without chloramphenicol. Chloramphenicol-susceptible colonies were subjected to colony PCR with primers flanking the IsaA gene, IsaAver_For (5'-GTGTTGATTGCTTTTTAATTGCG) and IsaAver_Rev (5'-CCAATTTCTATGGGAAGAGCT). Amplification of a ~1200 bp fragment indicated that the *isaA* gene was present while a smaller ~500 bp fragment indicated that *isaA* was deleted. To create AyrA(R233K)Δ*ayrBC* and IsaA(K2Q*)ΔayrRABC,* deletion plasmids previously created for Δ*ayrBC* and Δ*ayrRABC* (5) were introduced into AyrA(R233K) and IsaA(K2Q), and the above protocol was followed with successful deletions assessed by colony PCR using primers previously published (3).

Plasmid Construction. The plasmid pSK5630 was used to create all complementation vectors (6, 7). The template was genomic DNA isolated from *S. aureus* N315, unless otherwise stated. In all cases successful constructs were confirmed by DNA sequencing and plasmids introduced into N315 Δ*isaA* using electroporation as previously described (3).

P_{isaA}-*isaA* and P_{isaA}-isaA(K2Q) were created by PCR amplification of the *isaA* gene using genomic DNA from wildtype and the IsaA(K2Q) mutant strain, respectively. 400 bp upstream of the translational start site of *isaA* (to include the *isaA* promoter) was included using the primers IsaA_For (5'-ggaGGATCCGTGTTGATTGCTTTTTAATTGCG) IsaA_Rev (5'-TCCTCTAGACTAGTGATGGTGATGATGGTGAGAGCCTCCACCGAATCCCCAAGCACCTAAACC). A His_{x6} tag with a Gly_{x3}Ser linker was added to the C-terminus of *isaA* alleles to allow for Western blot analysis. The amplified products were introduced into pSK5630 through restriction digest (BamHI and SmaI) and ligation.

All chimeric constructs were created by overlap extension. P_{isaA}-*BlaZ* was created using primers IsaA_For and BlaZpromWT_Rev (5' - AATTACAATTAAAAATATTAACTTTTTCAAAGTAAAAATCCTCCAGTAATAATTG) amplified the 400 bp promoter region of *isaA* and primers BlaZFL_For (5' – CTTACAATTATTACTGGAGGATTTTTACTTTGAAAAAGTTAATATTTTTAATTG) and BlaZFL_Rev (5' – GGTTTAGGTGCTTGGGGATTCTAGtctagaGGGAA) amplified the ~850bp *blaZ* gene. To install the resistance conferring K2Q mutations primers IsaA_For and BlaZpromK2Q_Rev (5' – AATTACAATTAAAAATATTAACTGTTTCAAAGTAAAAATCCTC CAGTAATAATTG) were used to amplify the promoter region and BlaZK2Q_For (5' – CTTACAATTATTACTGGAGGATTTTTACTTTGAAACAGTTAATATTTTTAATTG) and BlaZFL_Rev to amplify the *blaZ* gene. In both cases primers IsaA_For and BlaZFL_Rev were used to join the two fragments. The resulting ~1250 bp PCR products were cloned into pSK5630 using restriction digest (BamHI and SmaI) and ligation.

P_{isaA}-$IsaA_{sp}BlaZ_{ec}$ and P_{isaA}-$IsaA(K2Q)_{sp}BlaZ_{ec}$ were created using the IsaA_For primer with IsaAsp_Rev (5' – ATATTTTTTTCTAAATCATTTAACTCTTTAGCGTGTGCTTGATGTCCT) using WT and isaA(K2Q)genomic DNA, respectively. The extracellular domain of BlaZ was amplified using IsaAsp_FOR (5' – GCAGCAGGTACAGGACATCAAGCACACGCTAAAGAGTT AAATGATTTAGAAAA) and BlaZhis_Rev (5' - TCCAAGCTTCTAGTGATGGTGATGATGG TGAGAGCCTCCACCAAATTCCTTCATTACACTCTT). To join the promoter/signal peptide and extracellular domain PCR products primers IsaA_For and BlaZhis_Rev were used for both WT and the K2Q mutant. The resulting PCR fragments were introduced into pSK5630 by restriction digest (BamHI and SmaI) and ligation. To install the non-cleavable proline mutation, the final constructs were subject to site directed mutagenesis using the primers Pro_For (5' - CACACGCTCCTGAAGTAAACGT) and Pro_Rev (5'- CTTGATGTCCTGTACCTGCTG).

All plasmids in which only the IsaA signal peptide was swapped were created using P*isaA*-isaA as the template and the following primers:
P_{isaA}-$SA1754_{sp}IsaA_{ec}$ SA1754WT_For (5' – GCTAAAACGATTGCTAAAGTT CCCGCAAGTATAGACTTTTTCATAGTAAAAAATCC) SA1754sp_Rev (5' – ATCACCACT AGTAACTAATCTAGATAAAAATGAGGCACAAGCTGCTGAAGTAAAC), P_{isaA}-$SA1754(K2Q)_{sp}IsaA_{ec}$ SA1754K2Q_For (5' – GCTAAAACGATTGCTAAAGTTCCCGCAAGTA TAGACTTTTGCATAGTAAAAAATCC) and SA1754sp_Rev, P_{isaA}-$SceD_{sp}IsaA_{ec}$ SceDWT_For (5' – AACCTACTGCTAATGATGATGCGAGTAATGTCTTTTTCATAGTAAAAAATCC) SceDsp_Rev (5' – TAGGAATCGTAGCAGGAAATGCAGGTCACGAAGCCCATGCA GCTGAAGTAAAC), P_{isaA}- $SceD(K2Q)_{sp}IsaA_{ec}$ SceDK2Q_For (5' – AACCTACTGCTAATGA TGATGCGAGTAATGTCTTTTGCATAGTAAAAAATCC) and SceDsp_Rev, and P_{isaA}- AtpF $(K2Q)_{sp}IsaA_{ec}$ and AtpFK2Q_For (5' – ACTCAACGCCTCCAGCTGCACCAAGAACGAATAA CTTTTGCATAGTAAAAAATCCTC) AtpFK2Q_Rev (5' –). The resulting ~7 kb linear full-length plasmids were DpnI treated overnight at 37 °C. PCR product (100 ng) was treated with T4 kinase for 30 min at 37 °C and ligated.

RNA isolation and RT-PCR. RNA isolation and conversion to cDNA was carried out as previously described (3). SYBR Select (Invitrogen) and a Bio-Rad CFX Connect real-time system were used for reverse transcription (RT)-PCR analysis using cDNA template (50 ng) and

the primers (625 nM) previously described in Table S2 of Craney, 2015 (3). The *gmk* gene was used to normalize gene expression and the parental strain *S. aureus* N315 was used to normalize gene expression between strains. Gene expression changes were measured in triplicate using the $\Delta\Delta C_T$ method and CFX Manager 3.0 software (Bio-Rad).

Western blot analysis. The secretion of IsaA and BlaZ His-tagged alleles were assessed by western blot analysis. Cells were grown to an OD_{600} of 0.6 in TSB plus chloramphenicol, pelleted by centrifugation and washed twice in sterile saline. After washing, the pellets were resuspended to an OD_{600} of 0.6 in TSB plus chloramphenicol and split in two 20 ml cultures with arylomycin M131 added to one and the other serving as the DMSO only control. Cultures were grown at 37 °C for 30 min and cultures were again pelleted by centrifugation. The supernatant was removed to a fresh 50 mL conical tube and subjected to TCA precipitation as previously described (8, 9). Samples were run on 15% SDS PAGE gels and transferred to PVDF for subsequent western blotting. For detection, an HRP-conjugated primary antibody directed at the 6× His epitope was used at 1:5000 (Proteintech, IL, USA) with membranes incubated for 1 h, washed 3× for 15 min in PBST and visualized using Pierce™ ECL Western Blotting Substrate (ThermoFischer Scientific, MA, USA), followed by exposure to Amersham Hyperfilm ECL film (GE Healthcare Life Sciences, PA, USA).

References

1. **Therien AG, Huber JL, Wilson KE, Beaulieu P, Caron A, Claveau D, Deschamps K, Donald RG, Galgoci AM, Gallant M, Gu X, Kevin NJ, Lafleur J, Leavitt PS, Lebeau-Jacob C, Lee SS, Lin MM, Michels AA, Ogawa AM, Painter RE, Parish CA, Park YW, Benton-Perdomo L, Petcu M, Phillips JW, Powles MA, Skorey KI, Tam J, Tan CM, Young K, Wong S, Waddell ST, Miesel L.** 2012. Broadening the spectrum of beta-lactam antibiotics through inhibition of signal peptidase type I. Antimicrob. Agents Chemother. **56:**4662-4670.
2. **Roberts TC, Smith PA, Cirz RT, Romesberg FE.** 2007. Structural and initial biological analysis of synthetic arylomycin A_2. J. Am. Chem. Soc. **129:**15830-15838.
3. **Craney A, Romesberg FE.** 2015. A putative Cro-Like repressor contributes to arylomycin resistance in *Staphylococcus aureus*. Antimicrob. Agents Chemother. **59:**3066-3074.
4. **Monk IR, Shah IM, Xu M, Tan MW, Foster TJ.** 2012. Transforming the untransformable: application of direct transformation to manipulate genetically *Staphylococcus aureus* and *Staphylococcus epidermidis*. MBio **3**.
5. **Craney A, Dix MM, Adhikary R, Cravatt BF, Romesberg FE.** 2015. An alternative terminal step of the general secretory pathway in *Staphylococcus aureus*. mBio **6**.
6. **Grkovic S, Brown MH, Hardie KM, Firth N, Skurray RA.** 2003. Stable low-copy-number *Staphylococcus aureus* shuttle vectors. Microbiology **149:**785-794.
7. **Stapleton MR, Horsburgh MJ, Hayhurst EJ, Wright L, Jonsson IM, Tarkowski A, Kokai-Kun JF, Mond JJ, Foster SJ.** 2007. Characterization of IsaA and SceD, two putative lytic transglycosylases of *Staphylococcus aureus*. J Bacteriol **189:**7316-7325.
8. **Powers ME, Smith PA, Roberts TC, Fowler BJ, King CC, Trauger SA, Siuzdak G, Romesberg FE.** 2011. Type I signal peptidase and protein secretion in *Staphylococcus epidermidis*. J. Bacteriol. **193:**340-348.
9. **Schallenberger MA, Niessen S, Shao C, Fowler BJ, Romesberg FE.** 2012. Type I signal peptidase and protein secretion in *Staphylococcus aureus*. J. Bacteriol. **194:**2677-2686.
